# A mass participatory experiment provides a rich temporal profile of temperature response in spring onions

**DOI:** 10.1002/pld3.126

**Published:** 2019-03-12

**Authors:** Anna Brestovitsky, Daphne Ezer

**Affiliations:** ^1^ Sainsbury Laboratory University of Cambridge Cambridge UK; ^2^ Alan Turing Institute London UK; ^3^ Department of Statistics University of Warwick Coventry UK

**Keywords:** citizen science, functional regression, mass participatory experiment, onion, spring onion, temperature, time series

## Abstract

Plants modulate their growth rates based on the environmental signals; however, it is difficult to experimentally test how natural temperature and light fluctuations affect growth, since realistic outdoor environments are difficult to replicate in controlled laboratory conditions, and it is expensive to conduct experiments in many environmentally diverse regions. In partnership with BBC Terrific Scientific, over 50 primary schools from around the UK grew spring onions outside of hydroponic growth chambers that they constructed. Over 2 weeks, students measured the height of the spring onions daily, while the hourly temperature and visibility data were determined for each school based on the UK Meteorological Office data. This rich time series data allowed us to model how plants integrate temperature and light signals to determine how much to grow, using techniques from functional data analysis. We determined that under nutrient‐poor hydroponic conditions, growth of spring onion is sensitive to even a few degrees change in temperature, and is most correlated with warm nighttime temperatures, high temperatures at the start of the experiment, and light exposure near the end of the experiment. We show that scientists can leverage schools to conduct experiments that leverage natural environmental variability to develop complex models of plant‐environment interactions.

## 1 INTRODUCTION

It is important for the general public to gain an appreciation and deeper understanding of the scientific process. However, outreach activities may take valuable time away from research. Citizen science provides a way for researchers to engage the public while collecting valuable scientific data that can augment their research.

Citizen science campaigns have been widely used for observational studies, such as the Big Garden Birdwatch (Devictor, Whittaker, & Beltrame, 2010). Alternatively, citizen science projects can be arranged so that participants are given specific microtasks, such as labeling telescope images (Fortson et al., 2011; Simpson, Page, & De Roure, 2014) or folding proteins (Cooper et al., 2010). Recently, the British Broadcasting Company (BBC) Terrific Scientific program launched a series of mass participation science experiments to get primary school students to collect data of scientific value. One example of the data collected as part of this project relates to the behavior of children, including their sleep patterns before and after daylight savings time and the influence of exercise on their attention spans. In another case, the data collected were related to schools, with data such as the local tree density, water quality, and electricity usage being collected.

In this study, we conduct a mass participatory experiment in primary schools and show that the data can be utilized to generate hypothesis that are testable in controlled laboratory settings. In particular, we focus on how plants integrate environmental signals.

Plants have complex molecular pathways that integrate light and temperature signals, both in response to brief temperature perturbations and in response to ambient temperature over longer time periods Nagano et al. (2019). In order for these molecular studies to have a direct impact on agricultural practice, we need to develop models to predict how temperature and light changes over time to influence crop growth. To build such a model, it is necessary to measure the growth under a wide range of natural environmental conditions, but data of this volume are difficult to access. For instance, researchers might only have access to data from sequential harvests on a small set of fields (Martre et al., 2018; Wu, Wang, Cheng, & Meng, 2016), and these fields might also be affected by confounding factors such as soil quality or plant pathogens. Alternatively, experiments can be performed under a large set of conditions in a controlled laboratory setting, but the conditions in growth chambers have difficulty mimicking natural temperature and light fluctuations. While there have been some attempts at developing growth chambers with more naturalistic diurnal changes (i.e., sinusoidal, rather than step functions), these cannot yet capture the full diversity of temperature and light fluctuations in natural environments (Annunziata et al., 2017, 2018).

One possible solution is to collect plant growth measurements from members of the public from diverse geographic regions, via citizen science.

We decided to team up with BBC Terrific Scientific to plan an experiment in schools to collect high‐resolution data about growth of spring onion over time in diverse outdoor environments. Spring onions were a good candidate for the experiment because height of spring onion is straightforward to measure and there are only a few major suppliers in the United Kingdom. We chose to run the experiment in a low‐nutrient hydroponic system to simplify the experimental protocol for the students thereby reducing experimental error. Furthermore, there is some interest in developing hydroponic growth systems for onions to improve taste and yield, and to provide more sustainable farming solutions in countries that have a dearth of arable land, or for use in urban agriculture (Treftz & Omaye, 2016).

Our mass participatory investigation enabled us to gather growth data from a wide range of natural environments. We found that primary school students were able to provide data that were sufficiently reliable to enable us to develop predictive models that were later confirmed in controlled laboratory conditions.

The data collected by the primary school students were also of sufficient quality to make a number of biologically relevant predictions, which are consistent with the literature and with follow‐up laboratory observations. Specifically, warm temperatures throughout the day are correlated with growth, with plants displaying a slightly elevated sensitivity to temperature at night. We also show that growth rates of spring onion throughout the time course are correlated with the temperature at earlier times during the time course.

## 2 MATERIALS AND METHODS

### 2.1 Ethical guidance and informed consent

Since the project involved young children (9‐ to 11‐year olds) and the data were accessed through a partnership with the BBC Terrific Scientific Program, we sought approval from the University of Warwick Ethics Board. As part of the informed consent processes, teachers needed to acknowledge that the survey data might be used for research purposes and that it might take up to 40 min to fill in the survey. Since no data were collected about the students, it was determined that they did not require informed consent from their parents. Teachers were reminded to make sure that none of their students were allergic to spring onions and to monitor the students when they used scissors.

### 2.2 Experiment performed by school children

**Figure 1:**
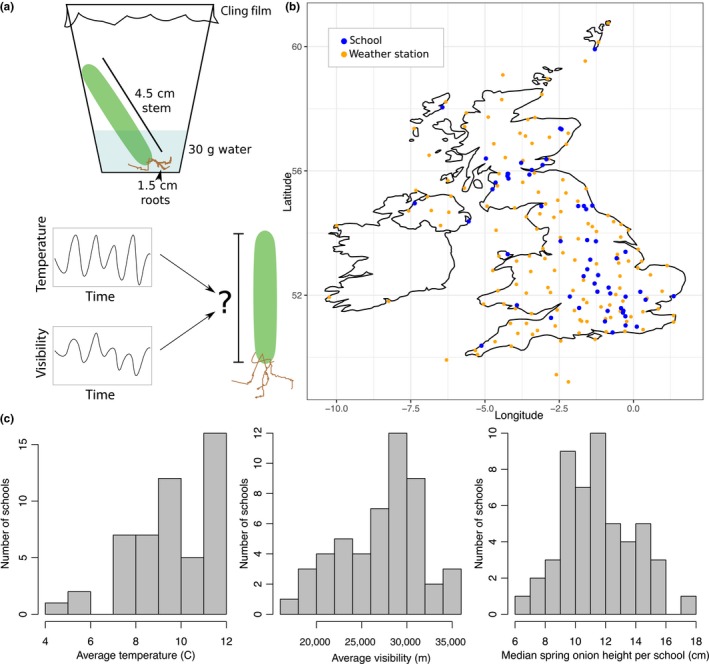
Set‐up of mass participation science experiment. (a) Students set‐up the growth controlled growth chambers for their spring onions from readily available materials. The goal of the project was to develop a model to predict the height of the spring onion using temperature and visibility measurements over time. (b) This map indicates the distribution of the UK weather stations and participating schools. (c) These histograms demonstrate the variability in average temperature, average visibility, and average spring onion height across the schools

Firstly, teachers recorded meta‐data about the spring onions, such as their country of origin and expiration date, since we did not have the resources available to distribute spring onions to the schools. On the first day of the experiment, students used a string to measure the circumference of the spring onion at its thickest point. Then, they trimmed the roots of the spring onion to 1.5 cm and trimmed the height to 4.5 cm. They placed the spring onions in growth chambers they constructed from 200 ml transparent plastic cups, 30 ml water, cling film, and a rubber band. Tape or blu tack was used to ensure that the spring onions stood upright (see Figure 1a). It was recommended that the growth chambers would be placed on the top of plastic school lunch trays that are usually standardized across state schools (since different surfaces might reflect/absorb heat differently). These chambers were placed outside, along one of the walls of the school, and a compass was used to determine the orientation of the wall. Every day for 2 weeks (excluding weekends), students recorded the height of the spring onions, measuring from the base of the stem and recording the time of day the measurement was taken (see Figure 1b). If the spring onions bent (as they sometimes do near the end of the time course), students were asked to stretch out the spring onion prior to measuring it. Students recorded their spring onion heights on individual tables and these were collated and uploaded via Qualtrics survey software by their teacher.

In order to ensure that the protocol was presented in a way that was easy for 9‐ to 11‐year‐old children to understand, we provided the BBC with the experimental protocol to translate into age‐appropriate written material and develop into a short demonstrative film. In total, 571 spring onions were grown in 57 schools, across a wide geographic range—from islands in north‐east and north‐west Scotland to the southern tip of England, as well as in schools in Wales and Northern Ireland (see Figure 1b).

### 2.3 Data processing and quality control of spring onion growth data

First, we looked for potential anomalies in the data, which might suggest data quality issues that could arise from recording mistakes made by teachers and/or students.

Two schools reported that they started the experiments on weekends. We have discovered that some schools run a weekend science club, but this nevertheless means that the tables are difficult to interpret since we specified weekday measurements and assumed there would be at most five measurements a week. One school reported that the expiration date was 13 days in the future, although it seems unlikely that supermarkets would stock spring onions with such far off expiration dates (other schools reported at most 5 days). This school was included in all the analysis, except when analyzing the affect of expiration date on spring onion growth.

There was one spring onion which was reported as being 25 cm, which is unrealistic growth in 2 weeks. An additional three spring onions grew more than 20 cm, which would mean that they grew taller than the plastic cups; it is unclear whether the spring onions pulled off the cling film or stretched it, and this might affect their growth rate. We decided to include these spring onions regardless, especially since many of the models were trained on the school's median height values.

Moreover, there were 16 cases in which the spring onions were reported to have shrunk by more than 1 cm over the course of a single day. In one case, this was clearly caused by a typing mistake—a digit was missing. In all but three of the other cases, the height drop appears within the last 4 days of the time course, when the height of the spring onions are more difficult to measure accurately due to bending of the spring onion stem. Fortunately, there were not more than two spring onions reported to have shrunk in any school, suggesting that this was not consistent error in a specific school. The measurement that appeared to be a typing error was replaced by a missing value. The other spring onions that were reported as shrinking were included in most of the downstream analysis. Since we only fit the fully functional regression model to the median heights this was not an issue, as the median heights were all monotonically increasing over time.

There were also data quality issues related to reporting of country of origin, since there is no standardized way of reporting this across supermarkets. For instance, some schools reported that the spring onions were from “Scotland,” the “UK,” and the “EU”. Since Scotland is in the United Kingdom which is currently still in the European Union, it could be that all these spring onions are from the same source, but this is not necessarily true. We decided to group all the United Kingdom‐based spring onions, and include the EU spring onions in the “other” category.

### 2.4 Access and data processing of meteorological data

Using the UK Met Office DataPoint API, we wrote a script that downloaded and extracted the hourly temperature and visibility data from each Met Office weather station. To predict the temperature and visibility at each school, we found the longitude and latitude coordinates that corresponded to the postal code of each school. We then interpolated between the weather stations using a thin plane spline regression with great circle distances, using the fields R package. An example of the interpolated temperatures is shown in Supporting Information Figure S7. Because the weather stations are not evenly distributed across the UK, the temperature predictions for schools in some geographic regions might be more reliable than others.

### 2.5 Statistical approaches

Traditional statistics methods aim to predict a scalar (or categorical)‐dependent variable, based on a set of scalar (or categorical)‐independent variables. In the present case, this would correspond to predicting the final height of the spring onion based on the minimum/maximum temperature, the average visibility, etc. However, some of the data we collected are inherently *functional*, for instance, the temperature as a function of time. It is possible to summarize this functional data by a scalar—for instance, taking the average temperature over the time course—but this loses the richness of the data. Functional regression produces predictive models of either scalars (final height of the spring onion) or functions (height of the spring onion over time) from a set of input functions (temperature or visibility over time). In this paper, we apply functional regression approaches (function‐to‐scalar or function‐to‐function regression) to predict the final spring onion height and the growth rates of the spring onions, respectively.

#### 2.5.1 Scalar‐to‐scalar and function‐to‐scalar models

Since there were significant batch effects, and there were different numbers of spring onions per school, we fit all models to the median spring onion height per school. We wished to select the most predictive model and to ensure that we were not overfitting our data, so our selection criteria were the model that minimized the leave‐one‐out cross‐validation score.

As a baseline, we calculated the leave‐one‐out cross‐validation score in the case where we assigned each spring onion to the mean height.

Next, we looked at six possible input variables: the average temperature or visibility over the 2‐week time course (scalar), the average temperature or visibility over a day (function over 24 hr), and the temperature or visibility over the entire time course (function over 360 hr). All models that considered one or two of these variables were considered. For the functional variables, two important pre‐processing steps were performed. Firstly, a *z*‐score was calculated at each time point, so that the average value and variability at each time point did not influence the outcome:

$$x_{t,i}^{*}=\frac{x_{t,i}-\text{mean}\left(x_{t}\right)}{\text{stdev}(x_{t})}$$

where $x_{t,i}^{*}$ is the *z*‐score for the data point *x*_*t*,*i*_ (which could be temperature or visibility) at time point *t* for school *i*.

Secondly, we needed to transform the tabular data into a function, which was done with a cubic B‐spline. (The scalar variables were expressed at constant functions over time, so that the same code could be used to calculate all values). After these pre‐processing steps, we tried to find *α* and *β* to fit the following form:

$$y=\alpha +\int \beta \left(t\right)x^{*}\left(t\right)dt$$

where *y* is the median spring onion height per school, x*(*t*) is a function of the *z*‐scores of temperature or visibility over time, *α* is the offset, and *β* is the weight. In the case when we looked at two variables at once:

$$y=\alpha +\int \beta_{1}\left(t\right)x_{1}^{*}\left(t\right)dt+\int \beta_{2}\left(s\right)x_{2}^{*}\left(s\right)ds$$

where $x_{2}^{*}\left(s\right)$ is the second variable under consideration over time, and *β*_2_(*s*) is the associated weight. *α* and *β*s were selected to minimize the least square error with an additional penalty term for smoothness of the *β*s. Specifically, the penalty term *p*_c_ was given by:

$$p_{c}=10^{\lambda }\int \frac{\delta^{\prime \prime }\left(\beta \left(t\right)\right)}{\delta t^{\prime \prime }}dt$$

*λ* is another free parameter and it was varied from 2–12 in increments of 0.1, and the value that minimized the leave‐one‐out cross‐validation error was selected. Note that, we also needed to choose a basis to represent *β*(*t*). For the temperature and visibility over the entire time course, we chose a B‐spline of degree 5, which was chosen since we expect that the weights will be spatially localized, and we needed a sufficiently high order B‐spline so that we could take the second derivative in order to calculate the penalty function. For the *β*s that provide a weight for the average temperature and light over the course of the day, we would like the value at either end of the function (which both represent midnight) to have the same value and slope, so we parameterize these in terms of a basis of Fourier functions with frequencies which are multiples of (one day)^−1^. For this entire analysis, we rely heavily on the fda R package (Ramsay, Hooker, & Graves, 2009; Ramsay & Silverman, 2005).

#### 2.5.2 Function‐to‐function model

To analyze the growth of the spring onions, we calculated the median height of the spring onions for each school, excluding schools that began their experiments on weekends. We fit the curves with monotonic cubic splines using the fda package and calculated the rate of growth over time from the fitted cubic spline (i.e., the first derivative of the monotonic cubic spline).

We used the FDboost package in R (Brockhaus, Melcher, Leisch, & Greven, 2017; Brockhaus, Scheipl, Hothorn, & Greven, 2015) to fit a model of the following form:

$$y\left(t\right)=\alpha +\int_{0}^{t}\beta_{1}\left(s,\;t\right)x_{1}\left(s\right)ds+\int_{0}^{t}\beta_{2}\left(s,\;t\right)x_{2}\left(s\right)ds$$

where the functional response variable *y*(*t*) is the rate of spring onion growth over time in each school. An important note in this model is that *s* is always less than *t*, so only past temperature can inform the current growth rate.

Children measured the spring onion heights whenever it was most convenient, so that this project would interfere with the school day as little as possible. This means that the time of day of data collection was not uniform across schools, and could even vary day‐by‐day within a school. Functional regression helps us handle the uneven time point sampling, because we can find the growth rate of the spring onions as a function of the *number of hours since experimental set‐up*.

### 2.6 Experiments performed in controlled growth chambers

The number of spring onions that could be tested in the laboratory was limited by the large amount of physical space they occupy, their expense compared to seeds, and the length of time for setting up each independent growth chamber. The latter variable is important, because we want to minimize the length of time between setting up the first and last spring onion to reduce variability arising from the amount of time the spring onions sat on the counter, rather than in the growth chamber. Even though we did not have the facilities to conduct large‐scale experiments of spring onion growth, we were still able to collect preliminary data that helped us to interpret the results acquired by the mass participation science experiment.

In order to test the protocol, we grew 63 supermarket‐purchased spring onions from eight batches (bags or bunches) that were selected from three different supermarket chains. These were grown in long‐day (LD) conditions (12 hr day, 12 hr night) at a constant 12°C. One of the supermarkets (two batches) only had spring onions that were pre‐washed and had trimmed roots, while the other batches were muddy and had roots of various sizes. To test the effect of root trimming, we trimmed half of the roots from each of the four batches that came from supermarket 3, but this had no effect on spring onion growth. The circumferences of spring onion were also measured.

After the mass participation science experiment was over, we grew an additional 66 spring onions to test some of the predictions made by the model. The spring onions came from 10 batches from the same supermarket, and each bunch was evenly distributed across the three experimental conditions. All the three experimental conditions were under LD conditions (note that in the UK the day length in March is approximately 12 hr, in agreement with LD conditions): the control had 10°C nighttime temperatures and 16°C day temperatures, the “warm day” conditions were at 10°C and 20°C, and the “warm night” conditions were at 14°C and 16°C. It is important to have colder nighttime temperatures than daytime temperatures in order to test realistic conditions. However, this also results in an additional confounding factor—we change both the pattern of temperature change over time and the size of the gap between the daily low and high, which might also affect growth.

In all cases, the spring onions were measured after 2 weeks.

## 3 RESULTS

### 3.1 School children can provide spring onion growth data of sufficient quality for modeling

Fifty‐seven schools throughout the United Kingdom grew spring onions outside for a period of 2 weeks as part of the experiment, with an average of about 10 replicates per school. The participating schools were widely distributed across the UK, included some in Northern Ireland, Wales, and Scotland (including the Shetland Islands and the Outer Hebrides) (Figure 1b). For the duration of the 2 weeks that the students grew the spring onions outside their school, we collected hourly temperature and visibility data from the MET Office weather stations and interpolated this to infer the hourly temperature and visibility at each school based on their supplied postcode.

**Figure 2:**
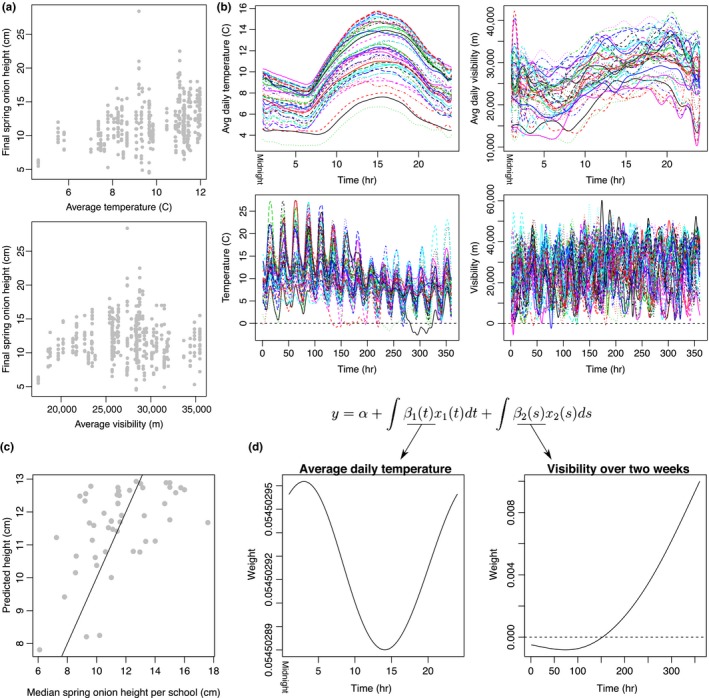
Functional regression of spring onion growth. (a) The heights spring onion measured by each student are shown in relation to the average temperature and average visibility observed at each school. (b) Each school (represented by each line) has a varied temperature and visibility profile over time. This shows the average temperature and visibility at each our of the day (top) and over the complete 2‐week experiment (bottom). (c) These are the results of the model that was most predictive of spring onion heights. (d) The form of the function and the associated weights are shown here. In this model, *x*_1_(*t*) corresponds to the average temperature at the time *t* during the day, and *x*_2_(*t*) corresponds to visibility at time *t* over the 2 weeks

There was a wide spread of temperatures, visibility measurements, and spring onion heights (Figure 1c), with a clear correlation between mean temperature and spring onion height. The same was not true for average visibility (an indicator of light) (Figure 2a), although this might be because the average temperature and visibility are not independent, with low visibility associated with both extreme high and low temperatures (Supporting Information Figure S1).

Prior to the start of the experiment, we tested whether spring onions purchased at different supermarkets had different growth rates under identical conditions and discovered that final spring onion heights were no more different than that would be expected by chance alone (Supporting Information Figure S2). Similarly, the preliminary analysis suggested that the initial circumference of the onions was not correlated with growth (Supporting Information Figure S3). For this reason, we let the teachers purchase spring onions from their local supermarkets.

Nevertheless, this preliminary result does not provide direct information regarding how spring onions from different sources or with different widths grow under *natural conditions*. We asked the schools to record various information such as the country of origin of the spring onions, the expiration date, and the circumference. With the exception of one school that did not report realistic spring onion circumferences, and four children who we presume forgot to include decimal places in their results, the students appeared to have measured the spring onion circumferences very accurately, producing a distribution of circumferences which was almost identical to the distribution we measured, suggesting that the students can take accurate measurements (Supporting Information Figure S3C). The students also reported no correlation between circumference and final spring onion heights, as expected.

The teacher's qualitative analysis of the amount of direct sunlight seemed to be slightly correlated with final spring onion height, but this effect disappears after controlling for temperature (Supporting Information Figure S4). However, this does suggest that schools in warmer areas also placed the spring onions in sunnier places, which might have exaggerated the temperature differences due to the greenhouse effect.

Other variables were also recorded by the schools, such as the country of origin of the spring onions, their expiration date, and the cardinal direction of the growth chambers in relation to the school building. Often the available data about the possible confounders was too sparse for a thorough mathematical treatment, but qualitatively they did not seem to have a substantial effect (Supporting Information Figure S5). These results suggest that the data were of sufficient quality for applying a functional regression approach. However, the results also highlight the importance of testing the predictions made by these models in a controlled laboratory setting.

### 3.2 Spring onion height is correlated with temperature and visibility in a time‐dependent manner

Although there is a clear correlation between average temperature and spring onion height, it is unclear whether this effect is time‐dependent (Figure 2a,b and Supporting Information Figure S5). Furthermore, studies on model organisms suggest that plants sense their environments at different time scales (Cortijo et al., 2017; Jung et al., 2016)—for instance, plants have different molecular mechanisms for sensing short‐term temperature fluctuations (on the scale of minutes) and longer‐term temperature changes (on the scale of days).

For this reason, we considered temperature and visibility, over three different temporal resolutions—their average value across the entire time course (constant basis), their average value at each hour of the day (Fourier basis), and their value over the 2‐week time course (B‐spline basis). This meant that there were six total input variables, and the four non‐constant input functions are shown in Figure 2b for each school. We considered all models that included up to two of these variables, and selected the model that was the best at predicting the height of the spring onion as determined by cross‐validation to prevent overfitting (Supporting Information Figure S6). The most predictive model was the one that included the average temperature over the course of a day and visibility over the entire time course, which substantially improved on the baseline cross‐validation error (244.00, as opposed to the 306.29 baseline) and had a Pearson's correlation of 0.52 (Figure 2c).

The weights attributed to the functional regression model suggest that nighttime temperature and the visibility at the end of the time course are the most correlated with the final spring onion height (Figure 2d). It is also important to note that temperature throughout the time course appeared to be positively correlated with the final height of the spring onion. Furthermore, the differences between the values of the weights in the night and during the day were small.

Among the models that only include one variable, the average temperature at each hour of the day is the most predictive (OCV of 245.46 and Pearson's correlation of 0.48). The temperature over the 2‐week period is most strongly correlated with the final spring height (0.50 alone and 0.59 when combined with two‐week visibility), but these models might over‐fit, as they produce worse results when cross‐validation is used to evaluate the models (see Supporting Information Table S1 for all comparisons of cross‐validation errors and Pearson's correlation).

### 3.3 Spring onions exhibit memory of temperature

**Figure 3:**
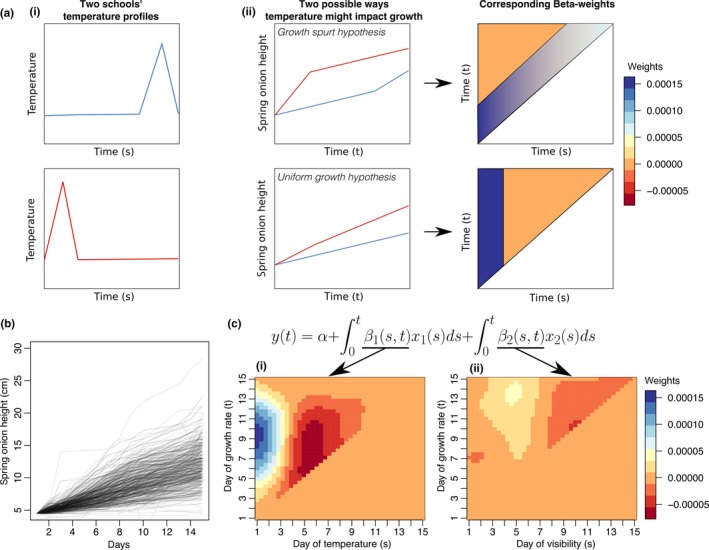
Fully functional regression suggests memory of early temperatures. (a) Suppose that there are two schools—one with an early burst of temperature and another with a late burst of temperature (i). One could imagine a growth spurt near the time where the temperature was elevated or one could image that the early temperature would affect the rate of growth throughout the 2‐week period. These types of patterns can be observed by looking at the weight matrix of a fully functional regression model (ii). In the heatmap illustrating the beta‐weights, the *x*‐axis represents the time in relation to the growth rate and the *y*‐axis represents the time in relation to the temperature. This forms a triangle, because temperatures at previous time points are potentially able to influence future growth rates, but the reverse is not true. (b) Qualitatively, the growth curves for the spring onions suggest near‐constant growth rates throughout the time course. (c) The fully functional model confirms that early temperatures are important in determining the growth rate for the entire time course

The functional regression analysis suggests that nighttime temperatures are predictive of the final spring onion height, but it is uncertain how early temperature affects spring onion growth rates *over time*. For instance, it is possible that early temperature spikes cause an early growth spurt in spring onion growths, but that growth rates become more uniform later in the time course. Alternatively, it may be that early temperature levels leave a lasting effect on the growth rates of spring onions throughout the time course (see Figure 3a).

Qualitatively, it appears that the spring onions have a relatively constant rate of growth throughout the time course, suggesting a model more similar to the second alternative presented above (see Figure 3b). To explore this more quantitatively, we fit a fully functional model to predict how temperature and visibility affect spring onion growth rates over time, which demonstrates that early temperature is correlated with the growth rates throughout the time course (see Figure 3c). Visibility barely had an effect on the measured growth rate, which might suggest that the correlation between visibility at the end of the time course and final spring onion height is a spurious effect (see Figure 3c). Overall, these results suggest that the spring onions have a *memory* of early temperatures that influences their growth rates for the duration of the experiment.

### 3.4 Experiments in controlled laboratory conditions are consistent with modeling output

**Figure 4:**
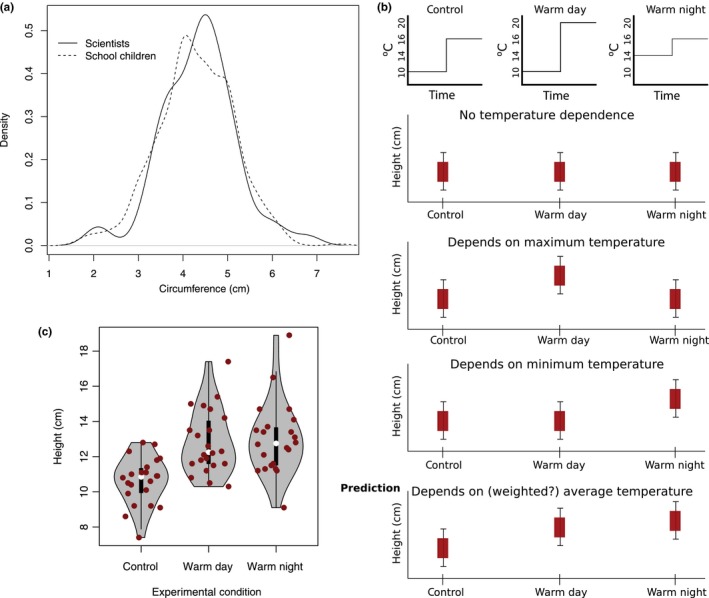
Experimental outcomes. (a) Primary school students produce a distribution of spring onion circumferences that is similar to the one measured by the authors. (b) An experimental set‐up was designed to help distinguish between a number of alternative hypotheses. The model based on the primary school data predicts the latter hypothesis. (c) The experimental results confirm the model

While our previous experiments demonstrated that the children could accurately measure the circumference of the spring onion (Figure 4a), we needed additional experiments to test whether the 2‐week long experiments provided reasonable results that could be replicated in controlled growth chambers.

Our models made a number of predictions, including that the height of the spring onion is affected by temperature throughout the day, but that it is slightly more affected by temperature at night (see Figure 2d). This model also predicts that the spring onions are sensitive to small changes in temperature—just a few degrees. For instance, the average temperature only varied by 8 degrees across the schools, but we still observed height differences among the spring onions (see Figure 2a).

This is in contrast to other possible models of how plants might integrate temperature to determine how much to grow. For instance, one might have supposed that maximal daytime temperature is the primary driver of spring onion growth. Alternatively, growth of spring onion might be rate‐limited by the lowest temperature (Figure 4b). We can distinguish between these hypotheses and the one presented by the model by growing the spring onion in conditions that mimic the average school temperatures. Then, we can test the effect of elevating the nighttime temperature by 4°C or the daytime temperature by 4°C.

We find that elevating nighttime or daytime temperatures causes a significant increase in spring onion height (both *p *< 0.0001 with a one‐tailed *t* test), so we can conclude that spring onions are sensitive to small temperature changes and that they do not exclusively depend on the daily maximum or minimum temperatures. Furthermore, the spring onions grown under the warm night condition were on average slightly taller than those grown in the warm day conditions, but this was not statistically significant, although it is consistent with the model. Furthermore, the model in Figure 2d suggests that the effect size is very small, so we would not expect to measure an observable difference given our limited sample size.

## 4 DISCUSSION

This study demonstrated that primary school students are capable of collecting rich datasets about plant growth that can be useful for modeling how plants integrate temperature and light signals. We determined that under low‐nutrient hydroponic conditions, growth of spring onion is proportional to temperature, particularly nighttime temperature (although the effect size is expected to be very small), which is consistent with the literature. Early in the time course, the growth rate depends on stored energy, as the students had cut off the majority of the green portion of the plant, but over time growth depends increasingly on photosynthesis. Finally, plants primarily grow at night, and the recently characterized PHYB‐EC‐PIF4 pathway seems to measure and respond to nighttime temperatures (Ezer et al., 2017; Jung et al., 2016; Legris et al., 2016).

Most of the molecular pathways describing the impact of light and temperature integration on growth have been performed on the dicot model organism *Arabidopsis thaliana* (Hayes et al., 2016; Jung et al., 2016; Legris et al., 2016; Pedmale et al., 2016), but it is important to determine whether these results extend to monocots, as these include some of the most important crops such as wheat, corn, and rice. Furthermore, our follow‐up experiments demonstrate that spring onion height can be affected by a 4 degree change in temperature during part of the day, demonstrating that spring onions have a high degree of temperature sensitivity.

Due to logistical constraints and the short term of the experiment, spring onions were grown from cuttings rather than from seeds, and they were grown in water rather than soil. In many ways this may be very different from how onions are usually grown in agricultural settings; however, there is increased interest in growing onions using hydroponics, either to enable more food to be produced in non‐arable regions, or to have finer‐grain control of nutrient uptake and taste (Treftz & Omaye, 2016). In future iterations of this project, it would be best if it was possible to distribute seeds and soil to schools, or at the very least distribute spring onions to the schools from the same suppliers. In addition, it would have been useful to record the temperatures within the growth chambers, as these might differ from the temperatures reported by the MET office due to proximity to the ground and the heat absorption properties of the materials of the chamber. However, in every citizen science project it is important for the research project to be accessible and simple, in order to recruit as many participants as possible, even though this sometimes has a detrimental effect on the quality of the data that can be collected. We think we struck a balance that enabled us to get a large number of schools to volunteer to participate in the research project, while also controlling for as many factors as were feasible. Critically, laboratory experiments partially confirmed the predictions made by the model trained on the data collected by the students.

From an outreach perspective, this project was successful at providing an educational science activity to schools that are located in remote locations, where it would normally be difficult for scientists to engage with schools. Furthermore, we demonstrated that the data were of sufficient quality for fitting a growth model suggesting that this is a promising way to merge outreach and research goals.

## CONFLICT OF INTEREST

The authors declare no conflict of interest associated with the workdescribed in this manuscript.

## AUTHOR CONTRIBUTIONS

AB and DE conceived and performed the experiment, analyzed the data, and wrote the manuscript.
