# Supplementary figures and images for: A mass participatory experiment provides a rich temporal profile of temperature response in spring onions
Source: Plant Direct. 2019 Mar 12;3(3):e00126. doi: 10.1002/pld3.126 (PMC6508787; doi:10.1002/pld3.126)

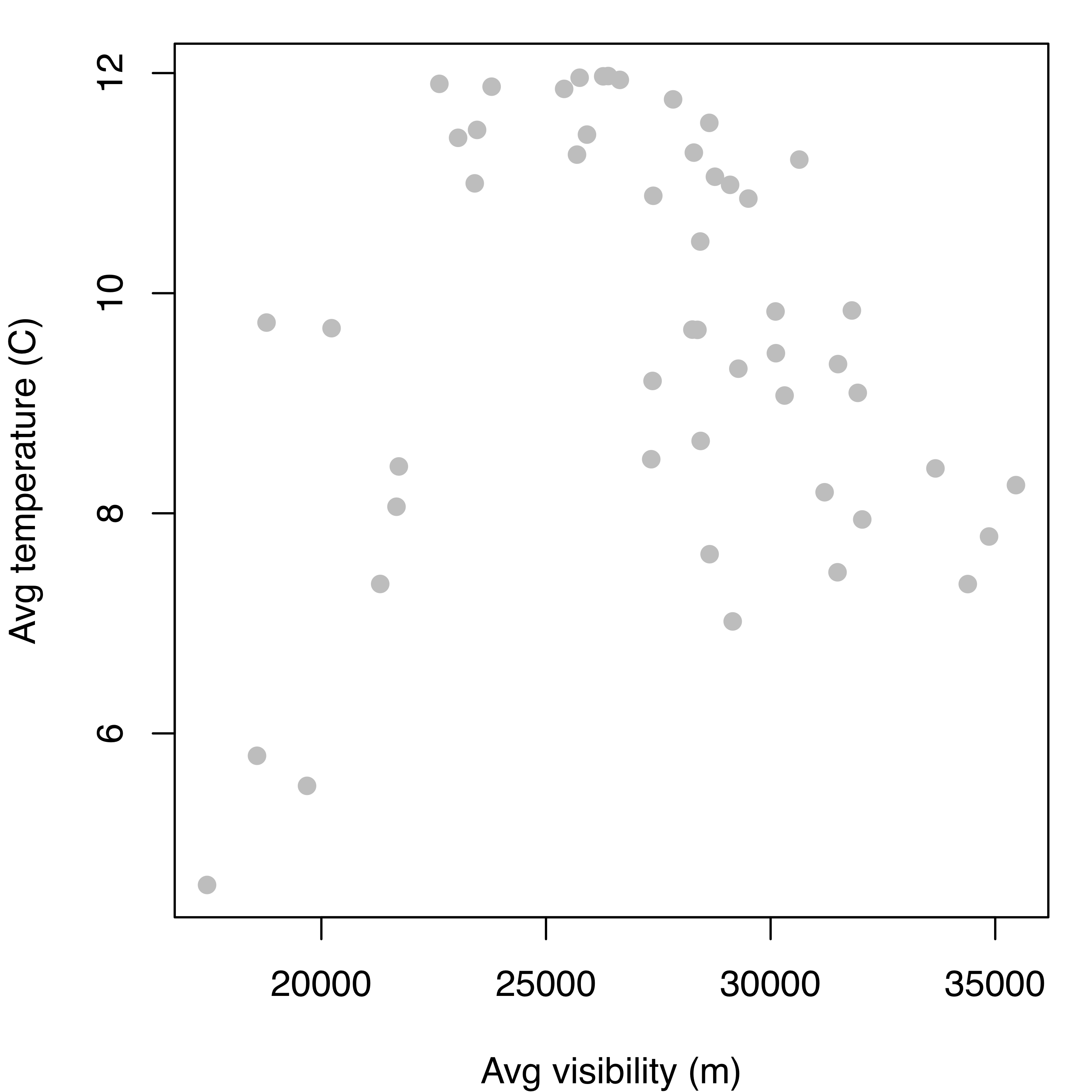

Supplement: Supplementary file 1 [file PLD3-3-e00126-s001.png]

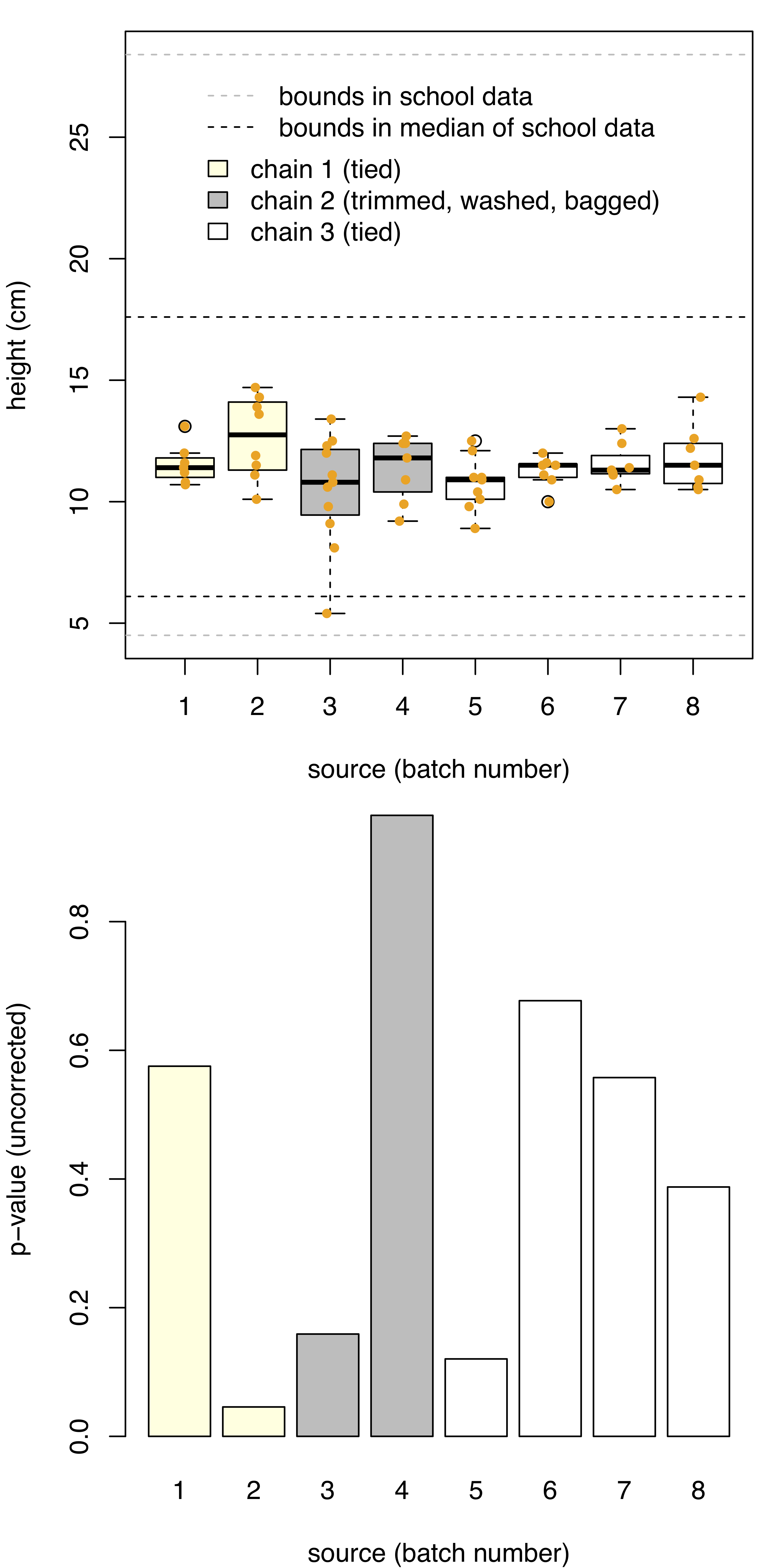

Supplement: Supplementary file 2 [file PLD3-3-e00126-s002.png]

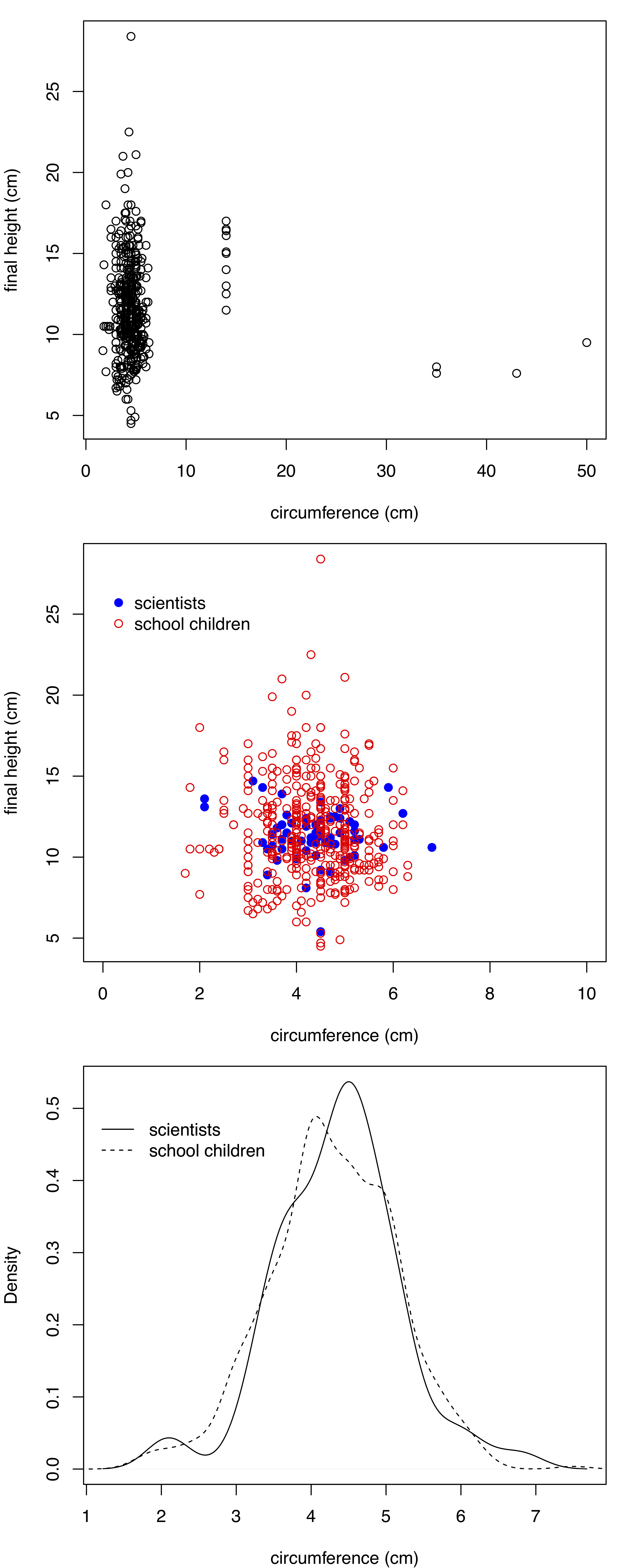

Supplement: Supplementary file 3 [file PLD3-3-e00126-s003.png]

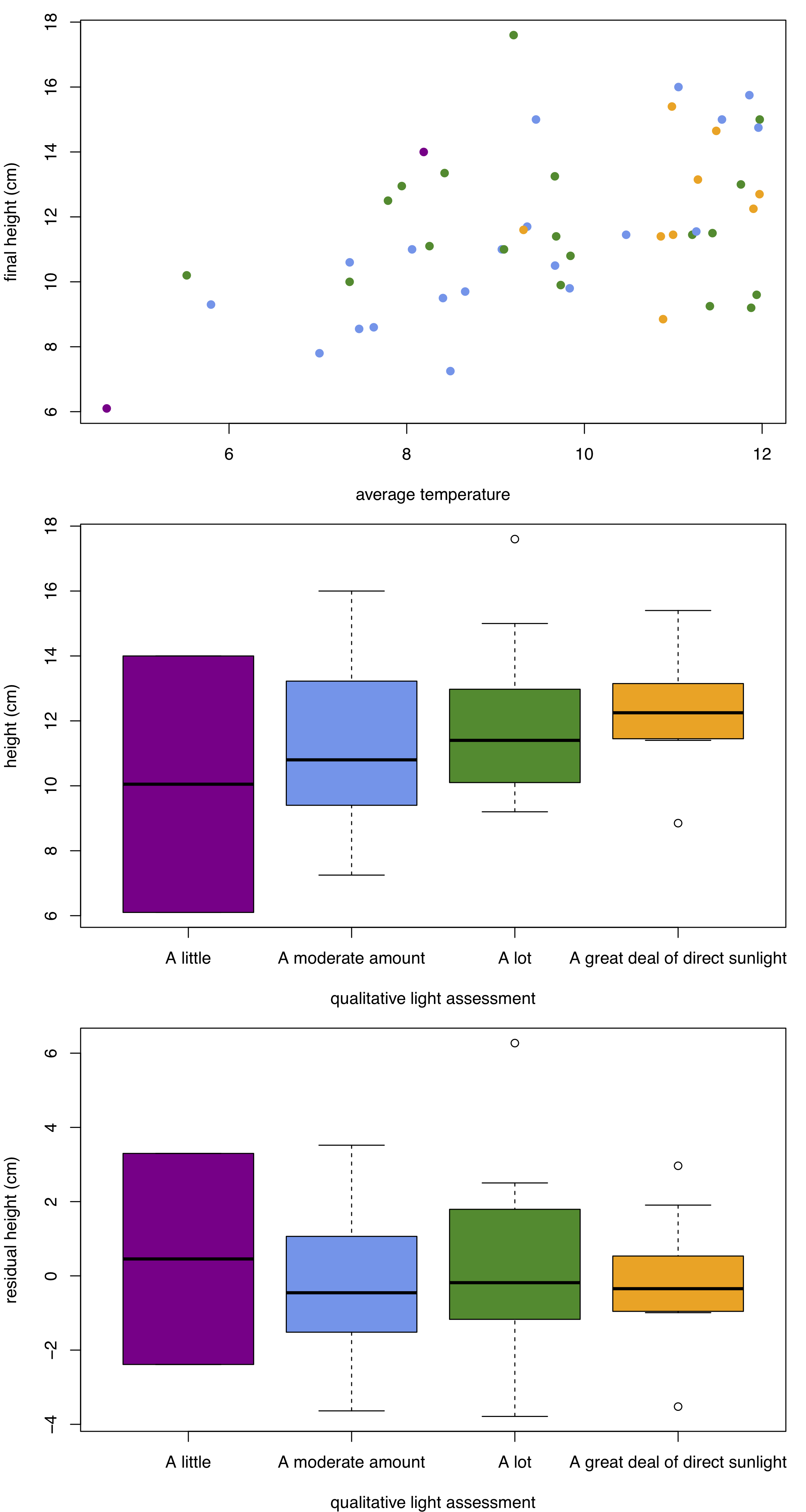

Supplement: Supplementary file 4 [file PLD3-3-e00126-s004.png]

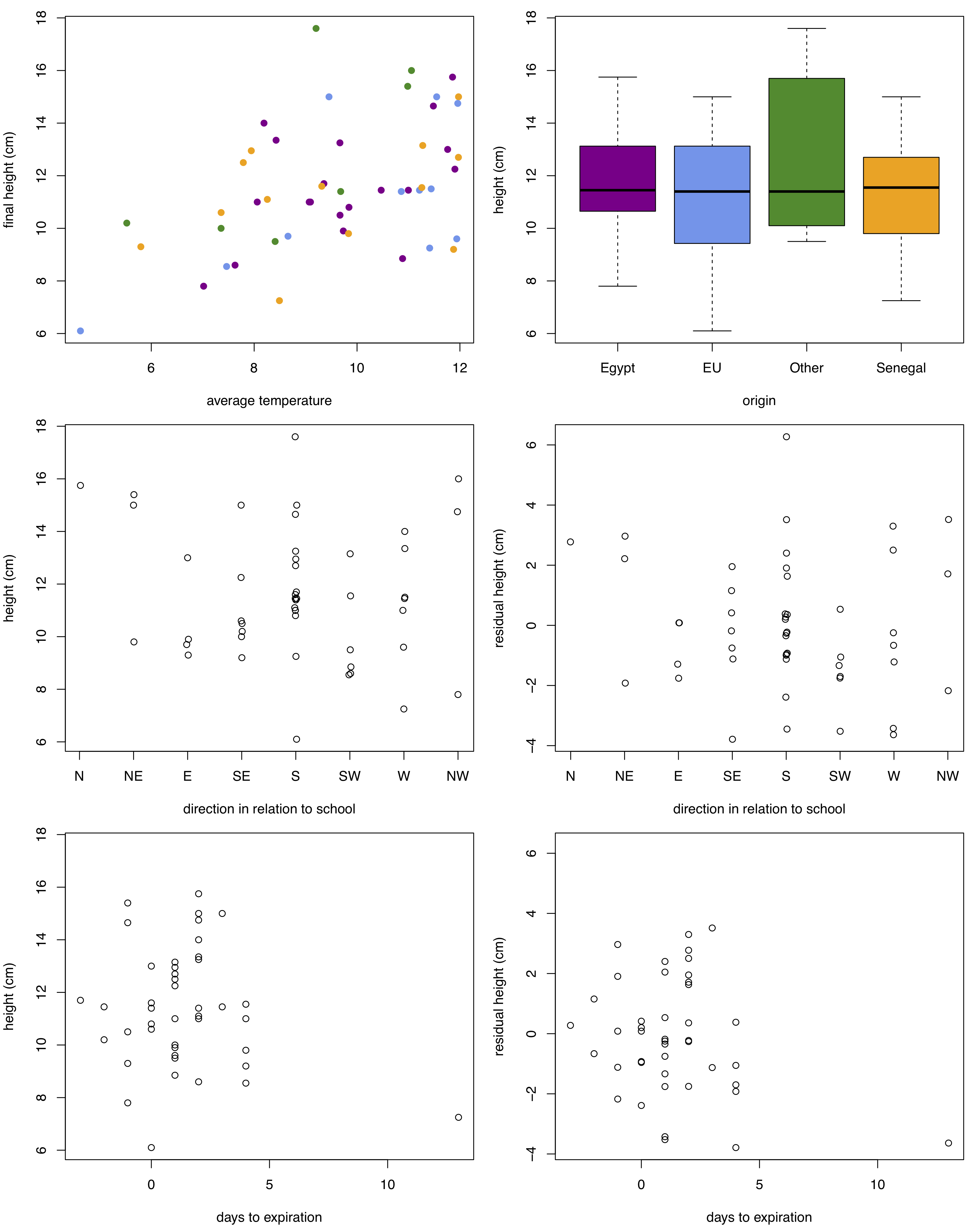

Supplement: Supplementary file 5 [file PLD3-3-e00126-s005.png]

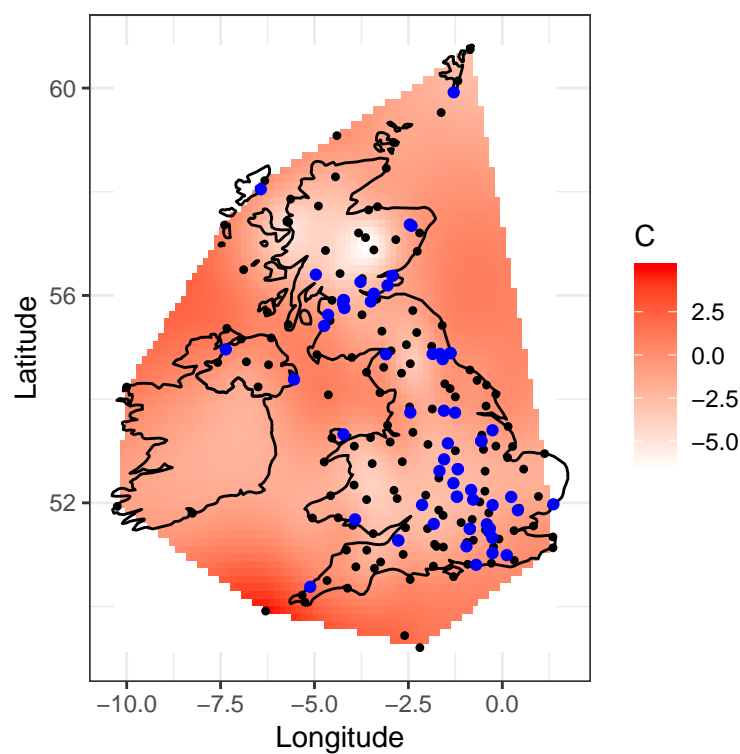

Supplement: Supplementary file 7 [file PLD3-3-e00126-s007.pdf]
