# Supplementary material for: A mass participatory experiment provides a rich temporal profile of temperature response in spring onions
Source: Plant Direct. 2019 Mar 12;3(3):e00126. doi: 10.1002/pld3.126 (PMC6508787; doi:10.1002/pld3.126)
Supplement: Supplementary file 6 [file PLD3-3-e00126-s006.pdf]

Figure S6: This compiles all of the regression models that were compared.

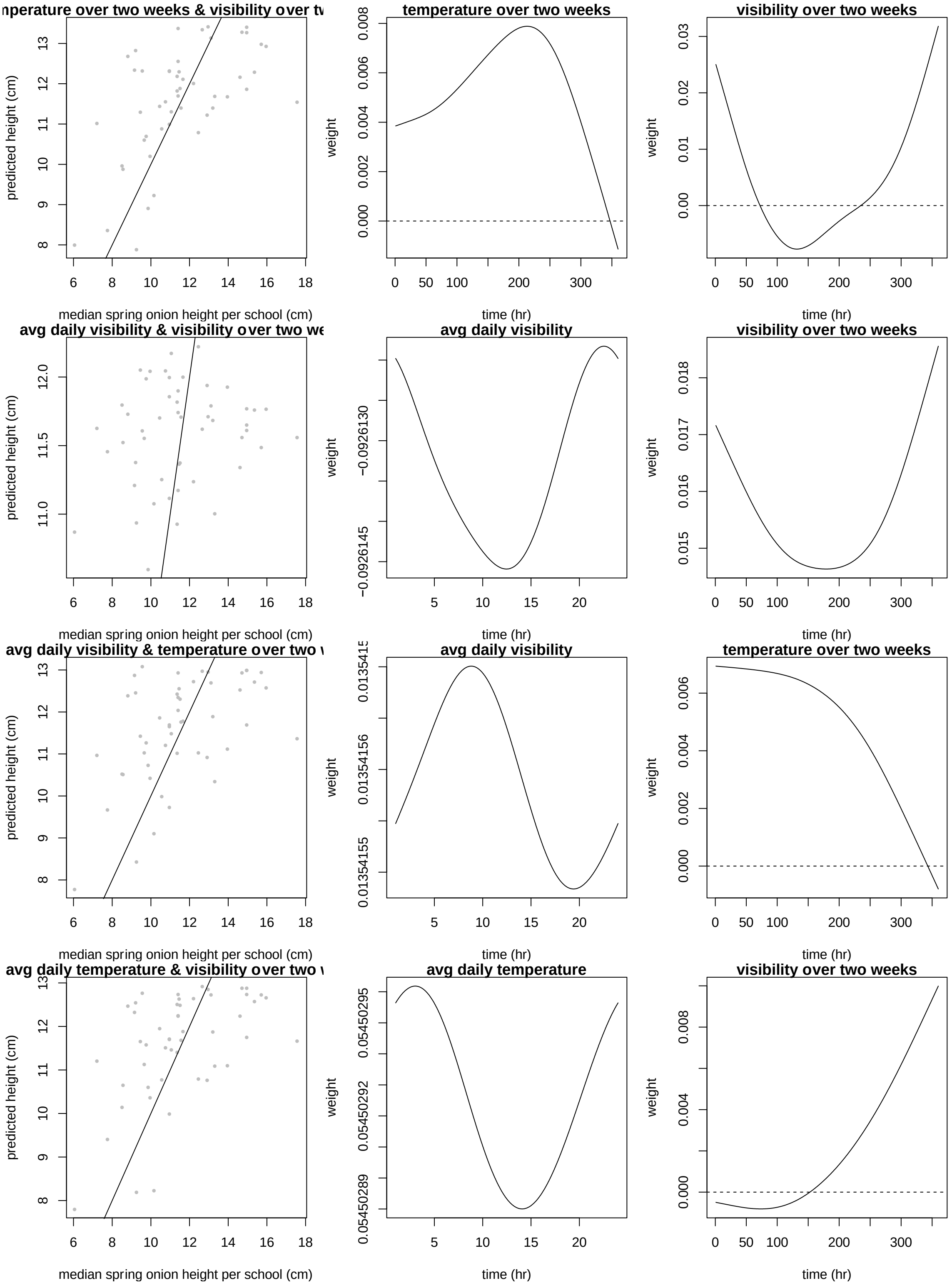

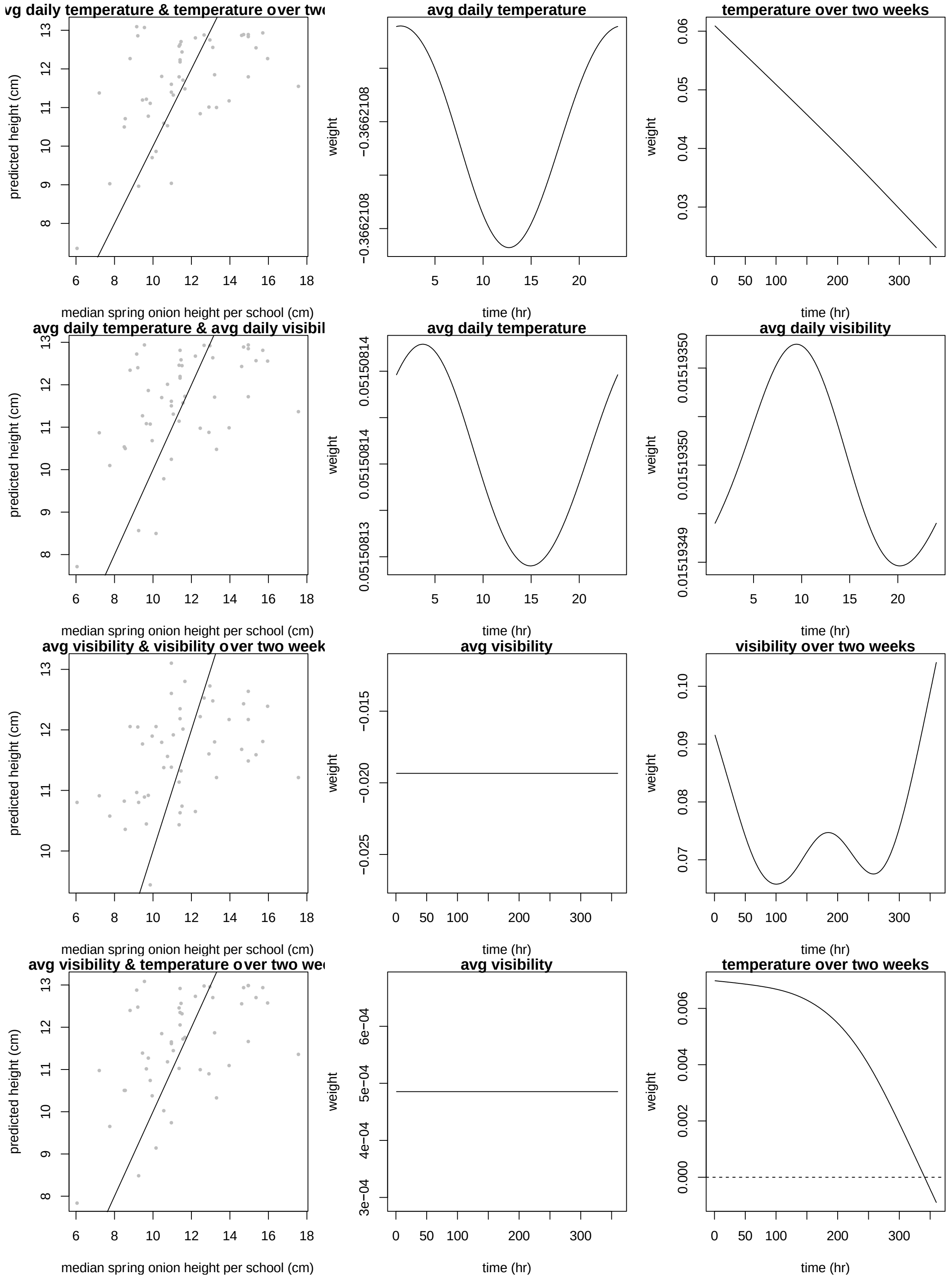
vg daily temperature & temperature over tw
predicted height (cm)
median spring onion height per school (cm)
avg daily temperature
weight
−0.3662108
time (hr)
temperature over two weeks
avg daily temperature & avg daily visibil
0.05150814
0.05150813
avg daily visibility
0.01519350
0.01519349
avg visibility & visibility over two week
avg visibility
−0.015
−0.020
−0.025
visibility over two weeks
avg visibility & temperature over two we
6e−04
5e−04
4e−04
3e−04

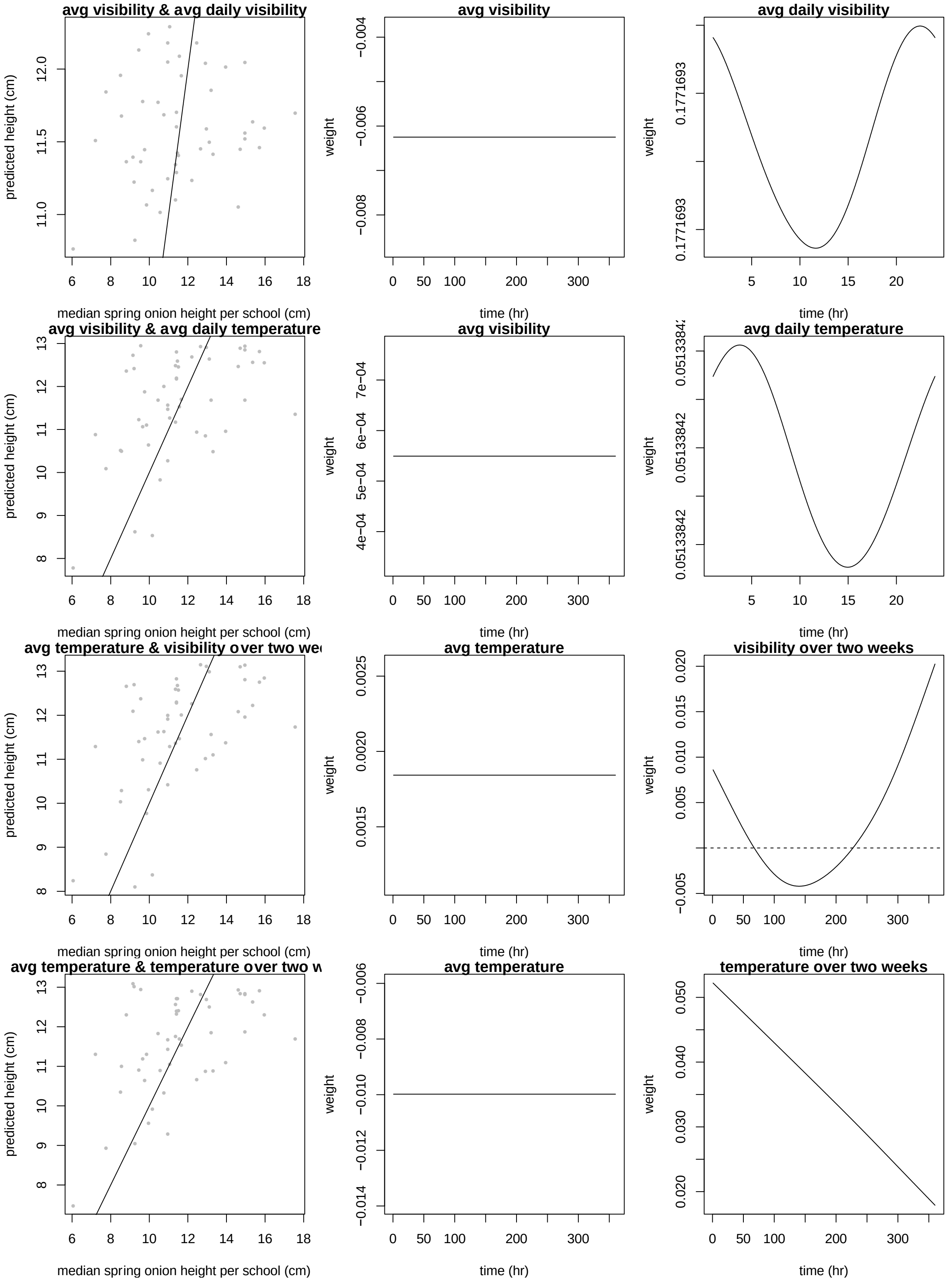
avg visibility & avg daily visibility
predicted height (cm)
median spring onion height per school (cm)
avg visibility
weight
time (hr)
avg daily visibility
0.1771693
0.1771693
avg visibility & avg daily temperature
avg visibility
7e−04
6e−04
5e−04
4e−04
avg daily temperature
0.0513384
0.0513384
0.0513384
avg temperature & visibility over two we
avg temperature
visibility over two weeks
avg temperature & temperature over two w
avg temperature
temperature over two weeks

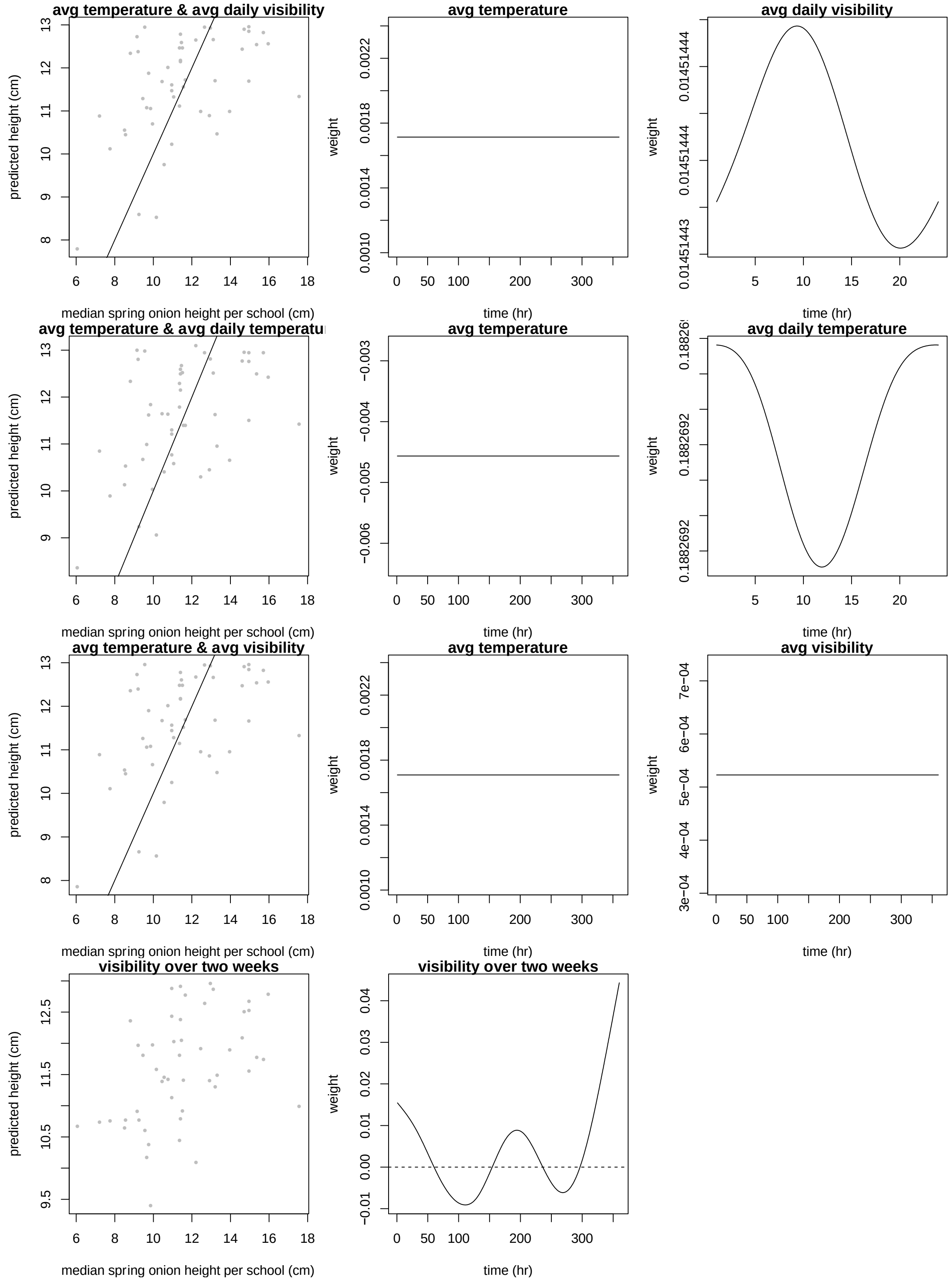
avg temperature & avg daily visibility
predicted height (cm)
median spring onion height per school (cm)
avg temperature
weight
time (hr)
avg daily visibility
weight
0.01451444
0.01451444
0.01451443
time (hr)
avg temperature & avg daily temperatu
predicted height (cm)
median spring onion height per school (cm)
avg temperature
weight
time (hr)
avg daily temperature
weight
0.1882692
0.1882692
time (hr)
avg temperature & avg visibility
predicted height (cm)
median spring onion height per school (cm)
avg temperature
weight
time (hr)
avg visibility
weight
time (hr)
visibility over two weeks
predicted height (cm)
median spring onion height per school (cm)
visibility over two weeks
weight
time (hr)

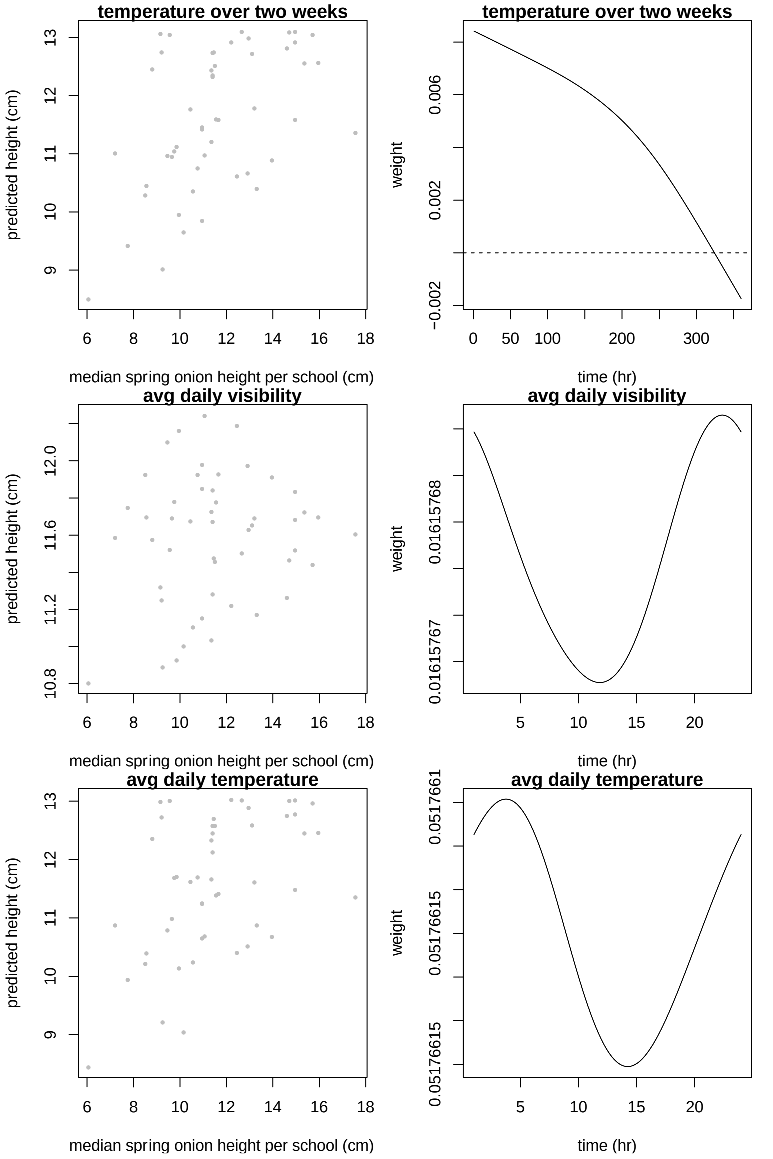
temperature over two weeks
predicted height (cm)
median spring onion height per school (cm)
temperature over two weeks
weight
time (hr)
avg daily visibility
predicted height (cm)
median spring onion height per school (cm)
avg daily visibility
weight
0.01615768
0.01615767
time (hr)
avg daily temperature
predicted height (cm)
median spring onion height per school (cm)
avg daily temperature
weight
0.0517661
0.051766615
0.051766615
time (hr)

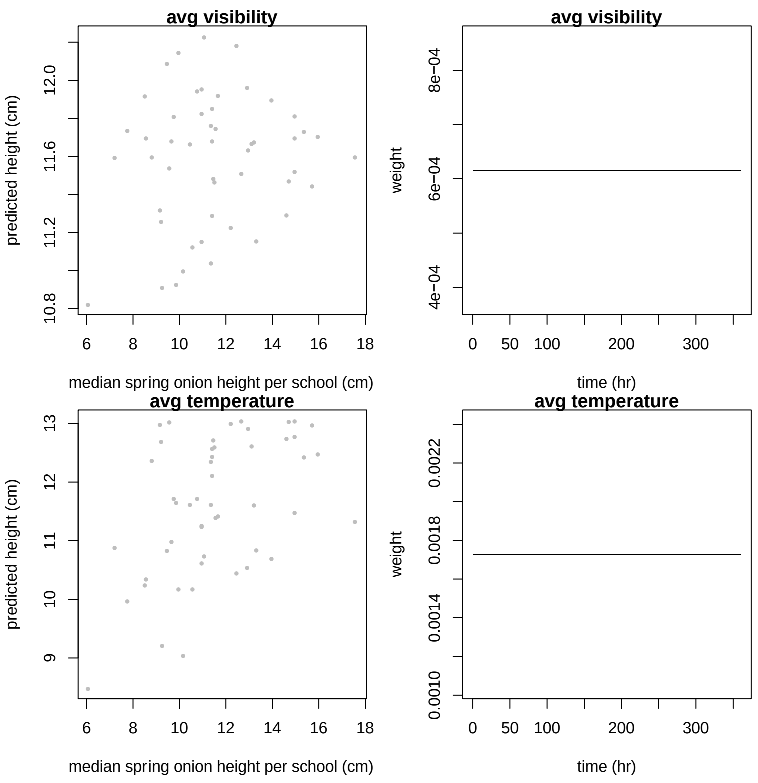
avg visibility
predicted height (cm)
median spring onion height per school (cm)
avg visibility
weight
time (hr)
avg temperature
predicted height (cm)
median spring onion height per school (cm)
avg temperature
weight
time (hr)
