# Supplementary material for: A mass participatory experiment provides a rich temporal profile of temperature response in spring onions
Source: Plant Direct. 2019 Mar 12;3(3):e00126. doi: 10.1002/pld3.126 (PMC6508787; doi:10.1002/pld3.126)
Supplement: Supplementary file 10 [file PLD3-3-e00126-s010.docx]

Figure S1: The relationship between average temperature and average visibility for each school.

Figure S2: Eight batches of spring onions were grown in 22°C in long day conditions. Two supermarket chains had their spring onions available in unpackaged bunches (tied), while one chain only provided spring onions that were pre-washed and had their root tips trimmed. In the top figure, the dashed lines represent the range of values that came from the BBC data, for comparison. In the bottom graph, the p-values from a student T-test for each batch, uncorrected for multiple hypothesis testing.

Figure S3: Here we compare spring onion circumferences to the final height of the spring onions. The top figure includes all data, but the outliers were removed in the bottom two figures, which provide some comparisons between the measurements taken by the primary school children and the authors of the paper.

Figure S4: This figure illustrates how the qualitative teacher assessment of the amount of direct sunlight (top) and how it is correlated to the final spring onion height (middle), but that this correlation disappears after controlling for the temperature (bottom).

Figure S5: A qualitative assessment of how country of origin, cardinal direction in relation to the school, and expiration date correlate with the final spring onion height.

Figure S6: This compiles all of the regression models that were compared.

Figure S7: This is an example of weather interpolation across weather stations at one time point. Weather stations are shown as black points and schools are shown as blue points.

Table S1: This table contains the data for the spring onion experiments in the growth chambers and from the primary school students (anonymised). The cross validation scores and Pearson's correlations of the models are also present.
