# Supplementary material for: A mass participatory experiment provides a rich temporal profile of temperature response in spring onions
Source: Plant Direct. 2019 Mar 12;3(3):e00126. doi: 10.1002/pld3.126 (PMC6508787; doi:10.1002/pld3.126)
Supplement: Supplementary file 9 [file PLD3-3-e00126-s009.docx]

Dear Tracy Lawson,

Thank you for the useful reviewer comments. Please consider our revisions for the manuscript. We think that we have addressed all of the reviewer concerns. Some of the changes we made include:

- Cutting back technical jargon and molecular biology from the introduction to improve flow
- Updating the labels and legend of Figure 3 to improve clarity
- Adding Ireland to Figure 1B for clarity.
- Improving the clarity of the language in a number of locations.

We think that these changes have substantially improved the quality of the manuscript. Thank you for re-considering our work and we look forward to submitting to Plant Direct in future.

Sincerely,

Daphne Ezer

Reviewer #1: 

This is an interesting study demonstrating how citizen science can be utilised to examine hypotheses generated in model organisms. 

Major comments 
1. Figure 3D is missing

**Oops—this is a typo on our part. It should have been 3C. This is now fixed.**

2. This is certainly my own ignorance, but I would personally appreciate a more approachable description of Figure 3A. How are time (s) and time (t) distinct?

**To clarify the difference between time (s) and time (t), we have added extra labels to Fig 3A. Furthermore, we have added more explanatory text to the legend:**

**In the heatmap illustrating the beta-weights, the x-axis represents the time in relation to the growth rate and the y-axis represents the time in relation to the temperature. This forms a triangle, because temperatures at previous time points are potentially able to influence future growth rates, but the reverse is not true.**

Minor comments 
3. I would appreciate further explanation of how meteorological conditions were extrapolated for each school- from the map it looks like there is considerable variation in the distance between each school and the closest weather station. How did the authors account for this?

**While we already mention the method we use for interpolating between weather stations (thin plane spline regression), we now provide (i) an example of what this looks like as a supplementary figure and (ii) a sentence describing how there are different levels of reliability in temperature measurements because of the uneven temperature sampling:**

**Because the weather stations are not evenly distributed across the UK, the temperature predictions for schools in some geographic regions might be more reliable than others.**

4. The methods note that students recorded the time of day they made their measurements- was this factored into the analysis?

**Yes, we do factor the time of day that the students recorded their measurements into the analysis. In fact, this is one of the benefits of functional data analysis, because it allows for analysis of datasets where the time points do not align. We emphasise this more clearly in the text:**

**Children measured the spring onion heights whenever it was most convenient, so that this project would interfere with the school day as little as possible. This means that the time of day of data collection were not uniform across schools, and could even vary day-by-day within a school. Functional regression helps us handle the uneven time point sampling, because we can find the growth rate of the spring onions as a function of the *number of hours since experimental set-up*.**

5. The authors note that growth in the field was dependent upon 'early' temperatures. Were the authors able to replicate this in their controlled environment setup?

**As our research group is computational, rather than experimental, we normally do not have access to growth chambers, but we were fortunate to be lent three chambers for a two-week period on two occasions (before and after the citizen science experiment)—see acknowledgements. Because of these limited resources, we were could only choose a single hypothesis from the citizen science experiment to test in controlled settings. We chose to test the circadian effect of temperature, because this corresponded with our prior research interests in circadian-dependent temperature response. However, we hope that by presenting the other results we would encourage researchers to test the effects of ‘early’ temperature memory.**

6. Typos throughout

**We have gone through and tried to fix as many typos we could spot.**

7. I was relieved to note that leaving the EU will at least provide further certainty into onion provenance in subsequent experiments. 

**This comment made us laugh.**

Reviewer #2: 

The work by Brestovitsky and Ezer is an ambitious crowdsourcing effort where elementary schools gathered data on onion growth, and then those measurements were compared to temperature and light data as determined by meteorological stations. The rationale is that something can be learned from analysis of large data sets regarding plant growth in natural environments, and that child labor may be exploited to drive scientific discovery. The authors then tested the results using controlled environments, and patterns observed were reproducible. 
Overall this is a nice piece of work that shows the value of citizen science as a data gathering tool. The fact that the work was taken full circle from models to chambers is good.

8. I question the logic of the writing and think the paper can be much improved with some revision that brings the reader in-this is not just creating new knowledge, this is showing a clever mechanism to do it. That's the hook, much more than a bigger onion during warmer nights.

**Thank you for your comments about the value of this ambitious crowdsourcing effort. We agree with you that we could have emphasised this aspect to a greater extent and have re-organised the introduction to address this, especially in response to Q12.**

Issues to address:

9. Figure 1B. It is strange that the experiments were done "across the UK, included some in Northern Ireland, Wales, and Scotland (including the Shetland Islands and the Outer Hebrides)" but the map in 1B only shows England, Scotland and Northern Ireland, completely detached from Ireland. A better map would be appreciated because this shows no context for the Irish experiments (and it looks weird).

**We have now added Ireland to the map to show the context more clearly.**

10. There is a lot of Discussion in the Results. The authors need to carefully check to not interpret the findings in the section.

**We have removed the entire paragraph beginning “These results are consistent with plant biology…”, as this is a Discussion point that we already discuss in the Results.**

11. it might be worth mentioning that the temperature and visibility data are likely not as accurate as they could be. Night temperatures will vary between cm and 2 m off the ground, and also are subject to many other topological considerations. It might be good to mention that. Incorporation of data from a web-based monitor or consensus of several stations (like Weather Underground etc) may help sharpen results. These are prevalent across the UK (and EU!)

**While we would have liked to use the Weather Underground, they no longer let new users access their weather API without a subscription. However, it is a very good point that the temperature within the growth chambers would not be the same as the average ambient temperature, as the material of the container, its proximity to the ground, etc, will affect the actual temperatures:**

**In addition, it would have been useful to record the temperatures within the growth chambers, as these might differ from the temperatures reported by the MET office due to proximity to the ground and the heat absorbtion properties of the materials of the chamber.**

12. The introduction dives into the molecular aspects of plant signal transduction when this paper is much more about ecophysiology. I would suggest revising the intro and maybe place the minutia of signal integration into the Discussion. The current intro is so bottom-up... we know this about signaling so how does it fit together? I think the more logical approach is, "Here is a model of how environment affects plant growth and here's how the molecular data support it."
    There is a lot of self-citation in the Introduction. I'd suggest adding additional references as well.

**We have removed the ecophysiology from the introduction, removed some of the self-referencing, and added a few more references about response to temperature fluctuations in natural conditions. Since these corrections affect the whole introduction, we do include the text in this response letter.**

13. The first paragraph of Results restates elements of the introduction.
    You can start with paragraph 2 without losing meaning.

**We have removed this paragraph**

14. Fix p<0.0001 on page 8

**Fixed**

15. Page 10 The data "were" not the data "was"

    **Fixed**


    ---------------------------------------------------------------------------- Editor comments:
16. Please try to streamline the overall structure of the the paper as suggested by reviewer 2. Also, please pay attention to the typos throughout the manuscript.
